# Health-related quality of life in young pediatric cancer survivors: The role of sarcopenia

**DOI:** 10.1007/s00520-026-10888-4

**Published:** 2026-06-15

**Authors:** Andres Marmol-Perez, Esther Ubago-Guisado, Cristina Cadenas-Sanchez, Andrea Rodriguez-Solana, Francisco J. Llorente-Cantarero, Juan Francisco Pascual-Gázquez, Daniel Moreira-Gonçalves, Jorge Mota, Kirsten K. Ness, Jonatan R. Ruiz, Luis Gracia-Marco

**Affiliations:** 1 Department of Physical Education and Sports, Faculty of Sport Sciences, Sport and Health University Research Institute (iMUDS), University of Granada, 18011 Granada, Spain; 2 Physical Exercise and Pediatric Cancer Research Group, Research Institute of Hospital, 12 de Octubre’ (‘imas12’), Madrid, Spain; 3 Department of Epidemiology and Cancer Control, St. Jude Children’s Research Hospital, Memphis, TN 38105 USA; 4 CIBER de Fisiopatología de La Obesidad y Nutrición (CIBEROBN), Instituto de Salud Carlos III, 28029 Madrid, Spain; 5 Cardiology Division, Veterans Affairs Palo Alto Health Care System, Palo Alto, CA USA; 6 Cardiology Division, Stanford University, Stanford, CA USA; 7 Instituto de Investigación Biomédica Maimonides (IMIBIC), 14004 Córdoba, Spain; 8 Departamento de Didácticas Específicas, Facultad de Educación, Universidad de Córdoba, 14071 Córdoba, Spain; 9 Servicio de Hematología y Oncología Infantil y del Adolescente. Unidad de Gestión Clínica de Pediatría y Cirugía Pediátrica. Hospital Universitario Virgen de Las Nieves, Granada, Spain; 10 Faculty of Sport, Research Center in Physical Activity, Health and Leisure, University of Porto, Porto, Portugal; 11 Laboratory for Integrative and Translational Research in Population Health, Porto, Portugal; 12 Faculty of Sport, University of Porto, Porto, Portugal; 13 Instituto de Investigación Biosanitaria Ibs.GRANADA, Granada, Spain

**Keywords:** Childhood cancer, Exercise, Muscular health, Rehabilitation, Well-being

## Abstract

**Purpose:**

Sarcopenia is a prevalent musculoskeletal complication after pediatric cancer, and its association with health-related quality of life (HRQoL) remains unknown. This study aimed to investigate health-related quality of life (HRQoL) differences according to sarcopenia status in young pediatric cancer survivors, and determined if these differences were distinct by sex.

**Methods:**

This cross-sectional study included 116 pediatric cancer survivors (12.1 ± 3.3 years old). Muscle strength was evaluated by handgrip strength test, while appendicular lean mass index (ALMI, kg/m^2^) was estimated via dual‑energy X‑ray absorptiometry. “Sarcopenia probable” was defined when muscle strength was ≤ decile 2 and ALMI Z-score was > -1.5 standard deviation (SD). “Sarcopenia confirmed” was defined when muscle strength was ≤ decile 2 and ALMI Z-score ≤ -1.5 SD. HRQoL was assessed using the Spanish version of the PedsQL 4.0 Generic Core Scales, and compared with age-specific reference values of healthy young population to calculate Z-scores.

**Results:**

Only female survivors without sarcopenia had significantly better total HRQoL (-0.3 [95% CI: -0.8 to 0.2] vs. 1.5 [95% CI: -2.1 to -0.8]), physical HRQoL (-0.3 [95% CI: -0.8 to 0.2] vs. -1.6 [95% CI: -2.3 to -0.9]), psychosocial HRQoL (-0.2 [95% CI: -0.7 to 0.3] vs. -1.2 [95% CI: -1.8 to -0.6]) and school HRQoL (-0.1 [95% CI: -0.6 to 0.4] vs. -1.3 [95% CI: -2.0 to -0.7]) than female survivors with sarcopenia confirmed.

**Conclusion:**

Most HRQoL domains are similar across sarcopenia status in young pediatric cancer survivors. However, female survivors without sarcopenia seem to have better HRQoL Z-score than those with sarcopenia.

**Supplementary Information:**

The online version contains supplementary material available at 10.1007/s00520-026-10888-4.

## Introduction

Survival following a cancer diagnosis in children has considerably increased over the past 60 years in developed countries with 5-year survival now exceeding 85% [1]. Survivors of pediatric cancer often report symptoms associated with treatment modalities which negatively affect their health-related quality of life (HRQoL) [2]. In this sense, HRQoL comprises a multidimensional construct including physical, mental, emotional, social, and behavioral components [3], and it is currently considered as an independent prognostic indicator of survival [4]. However, prior investigations have primarily targeted adult survivors. Young pediatric cancer survivors may provide valuable information to improve communication and shared decision making between clinicians and patients/caregivers about side effect recognition, management, and supportive care, with the goal of maximizing HRQoL [5].

Early exposure to aggressive cancer treatments (i.e., chemotherapy and/or radiotherapy) place young pediatric cancer survivors at high risk of reduced lean muscle mass and dysfunction [6] (hereafter referred to as sarcopenia)[7]. Sarcopenia is nowadays recognized as a significant public health concern among both adults and children [8–10]. The diagnosis of sarcopenia is associated with lower HRQoL in both adult cancer survivors [11] and adult pediatric cancer survivors [6]. However, the literature describing these associations in younger survivors is scarce. It remains unknown whether young pediatric cancer survivors without sarcopenia have better HRQoL than those with sarcopenia. In addition, prior studies have combined both female and male survivors in their analyses. This underscores the importance of conducting separate analyses for females and males to tailor sex-specific interventions and care plans in a more individualize manner.

The aim of this study was to evaluate HRQoL differences according to sarcopenia status in young pediatric cancer survivors, and determined if these differences were distinct by sex. We hypothesized that young pediatric cancer survivors without sarcopenia would likely have better HRQoL, especially female survivors who are at higher risk of low HRQoL than male survivors.

## Methods

### Study design and participants

This cross-sectional study included 116 young pediatric cancer survivors from the baseline data collection of the iBoneFIT project framework [12]. Survivors were enrolled from the Units of Pediatric Oncology and Hematology of the ‘Virgen de las Nieves’ (Granada) and ‘Reina Sofia’ (Cordoba) University Hospitals. Inclusion criteria were children previously diagnosed with cancer, at least one year from diagnosis, age 6 to 18 years, no under treatment, and prior exposure to radiotherapy and/or chemotherapy. Evaluations were carried out in two separate waves due to COVID-19 restrictions: first, from October to February 2020/2021; and second, from December to March 2021/2022. Parents and survivors signed informed consent and assent before starting the trial, respectively. The iBoneFIT project was approved by the Ethics Committee on Human Research of Regional Government of Andalusia (Reference: 4500, December 2019), followed the ethical guidelines of the Declaration of Helsinki (revised version 2013), and the randomized controlled trial was registered (https://www.isrctn.com/ISRCTN61195625). The Strengthening the Reporting of Observational Studies in Epidemiology (STROBE) checklist was used to report this study (Table S1).

### Anthropometry and somatic maturity

Body mass (kg) was evaluated with an electronic scale (SECA 861, Hamburg, Germany) with an accuracy of 100 g. Stature (cm) was assessed using a precision stadiometer (SECA 225, Hamburg, Germany) to the nearest 0.1 cm. Body mass index was calculated as body mass (kg)/stature (m^2^). In addition, age- and sex-specific body mass index Z-score and categories were calculated using international reference data for pediatric population according to the World Obesity Federation [13]. Somatic maturity was assessed by estimating the number of years before or after peak height velocity, using validated algorithms specific to boys and girls [14].

### Clinical data

Medical records were reviewed to obtain information on diagnosis, the interval between treatment completion and baseline data collection, and treatment exposures (including radiotherapy, chemotherapy, corticosteroids, and surgery, either individually or in combination). Time from treatment completion (in years) was analyzed as a continuous variable, while treatment exposures were categorized as dichotomous variables (yes/no).

### Physical activity

Total physical activity (min/day) was measured using tri-axial ActiGraph wGT3x-BT accelerometers (ActiGraph GT3X, Pensacola, FL, USA) worn for seven consecutive days (24 h/day). Survivors were instructed to wear the device on their non-dominant wrist, excluding during water-based activities. The accelerometers were initialized with a sampling frequency of 90 Hz, and the raw data were processed using the GGIR open-source R package (version 2.8–2) [15]. The Euclidean Norm Minus One (ENMO) of the raw acceleration, where values below one gravity were rounded to zero, was calculated, along with the z-axis angle of the device, to estimate physical activity and sleep parameters [16]. Non-wear time was identified based on the standard deviation of raw acceleration data across the three accelerometer axes, following previously described methods [17], and subsequently imputed using acceleration data from the same time window on the remaining days. Appropriate thresholds were applied to classify physical activity intensities (i.e., Moderate-to-vigorous physical activity: 200 mg and light physical activity: 35–200 mg) [18]. We considered a day valid when: 1) the accelerometer registered at least 23 h/day and 2) survivors wore the accelerometers at least 16 h/day since in this study the accelerometers were worn at both day and night [19].

### Dual‑energy X‑ray absorptiometry

Survivors were assessed using a single DXA scanner (Hologic Series Discovery QDR, Bedford, MA, USA), with analyses performed using APEX software (version 4.0.2). Daily calibration of the device was conducted using a lumbar spine phantom. Participants were instructed to lie still in the supine position during the scan, following the guidelines of the International Society for Clinical Densitometry. The total body DXA scan was used to obtain fat mass of the total body and lean mass (kg) [body mass – (fat mass + bone mass)] of the arms and legs (appendicular lean mass).

The appendicular lean mass index (ALMI, kg/m^2^) was calculated by dividing appendicular lean mass by height squared. All DXA scans were analyzed by a single trained researcher. The coefficient of variation for DXA in the pediatric population ranges from 1.0% to 2.9%, depending on the anatomical region [20]. Using international reference data from the Bone Mineral Density in Childhood Study, age-, sex- and race-specific total body fat mass and ALMI Z-score were calculated for analyses [21]. Survivors with ALMI ≤ −1.5 SD were classified with low ALMI, following prior reports in pediatric cancer survivors [22].

### Muscle strength

Handgrip strength (as an indicator of upper-body muscle strength) was assessed using a dynamometer (TKK 5101 Grip D, Takei, Tokyo, Japan). This test is both valid (intraclass correlation coefficients [ICC] ranging from 0.73 to 0.91) and highly reproducible (ICC between 0.91 and 0.93) [23]. Survivors performed two five-second maximal grip trials with each hand while keeping the arm extended and the highest value from each hand (in kilograms) was averaged for analysis [24].

To contextualize muscle strength in our sample, results were compared to updated age- and sex-specific reference values derived from nearly eight million test results from 34 countries, compiled by the FitBack network [25]. Muscle strength deficits were defined as values below the 2nd decile, in accordance with previously established sex- and age-specific percentile thresholds for fitness deficits by Tomkinson et al. [26].

### Sarcopenia

Sarcopenia status definition was followed according to the European Working Group on Sarcopenia in Older People (EWGSOP2)[27], in line with prior reports in this population [22]. “No sarcopenia” was defined when muscle strength was > decile 2. “Sarcopenia probable” was defined when muscle strength was ≤ decile 2 and ALMI Z-score was > −1.5 standard deviation (SD). “Sarcopenia confirmed” was defined when muscle strength was ≤ decile 2 and ALMI Z-score ≤ −1.5 SD. Since the EWGSOP2 cut-off values are based on adult populations, we used international reference data from healthy youth, as previously described [28], to assess muscle strength and ALMI.

### Health-related quality of life (HRQoL)

HRQoL was evaluated using the age-appropriate Spanish version of the 23-item self-report questionnaire, PedsQL 4.0 Generic Core Scales [29], which has been shown to be reliable and valid for pediatric cancer survivors [30]. Children aged 6–8 years received assistance from their parents to respond if needed, but parents did not complete the responses. This questionnaire uses a 5-point Likert scale (0 = Never a problem; 1 = Almost never a problem; 2 = Sometimes a problem; 3 = Often a problem; 4 = Always a problem) to assess how much each item has affected the survivor over the past month. PedsQL 4.0 includes four subscales: physical functioning (8 items), emotional functioning (5 items), social functioning (5 items), and school functioning (5 items). Items were reverse scored and linearly transformed to a 0–100 scale (0 = 100, 1 = 75, 2 = 50, 3 = 25, 4 = 0), with higher scores reflecting better HRQoL. Scale scores were calculated by averaging the responses, provided that at least 50% of the items in a subscale were completed; otherwise, the subscale score was not computed.

Three summary scores were derived: a total health summary score (based on all 23 items from the physical and psychosocial functioning subscales), a physical health summary score (based on the 8 physical functioning items), and a psychosocial health summary score (based on the 15 items from the emotional, social, and school functioning subscales). Higher scores indicate better perceived HRQoL. Finally, we compared each HRQoL domain (i.e., total score, physical health, psychosocial health, emotional, social and school functioning) with age-specific reference values of healthy young population to calculate Z-scores [31].

### Statistical analysis

The distributions of the variables were examined and confirmed using skewness and kurtosis values, the Kolmogorov–Smirnov test, and visual inspection of histograms, Q-Q plots, and box plots. Descriptive data were presented as means with standard deviations (SD) or as frequencies with corresponding percentages.

All results were stratified by sex because the two-way analysis of variance showed an interaction between sarcopenia status and sex for total (*P* = 0.024), psychosocial (*P* = 0.026) and school (*P* = 0.037) HRQoL Z-score. Analysis of covariance was used to test HRQoL Z-score differences (outcome variable) according to sarcopenia status. This analysis was adjusted for time from treatment completion, radiotherapy exposure, total body fat mass and total physical activity. To identify the minimum sufficient adjustment set (MSAS) for the differences in HRQoL Z-score according sarcopenia status, we built a theoretical causal diagram based on previous associations with muscle strength, lean mass and/or HRQoL available in the scientific literature [6, 28, 32, 33]. Given the collinearity between cancer diagnoses and related treatments, we built separate models. Considering the different impact of cancer treatments on sarcopenia and HRQoL, we included radiotherapy exposure due to its correlation with both sarcopenia and HRQoL (Table S2). We used the online tool DAGitty to construct a directed acyclic graph (DAG). The covariates time from treatment completion, radiotherapy exposure, total body fat mass and total physical activity were identified as the MSAS (Figure S1). Statistical analyses and figures were performed using the statistical software R version 4.4.0 (R Foundation for Statistical Computing) and *P*-values < 0.05 were considered statistically significant.

## Results

A total of 196 young pediatric cancer survivors were initially approached for participation (Figure S2). After inclusion/exclusion criteria screening, 116 survivors were enrolled and included in this study. Descriptive characteristics of the sample are shown in Table 1. Survivors had a mean (SD) age of 12.1 (3.3) years, 42.2% were female and the majority were diagnosed with blood cancers (60.9%). Nearly half of our sample did not have sarcopenia (43.1%), and this proportion was lower in females than males (40.8% vs. 44.8%, respectively). We did not observe considerable differences in HRQoL domains between female and male survivors, yet both reported lower HRQoL Z-scores than the normative reference values (Table 1).

**Table 1:** Descriptive characteristics of the survivors included in the study

Characteristics	Total	N	Females	N	Males	N
Sex (female, %)	42.2	116				
Age (years)	12.1 ± 3.3	116	12.2 ± 3.5	49	12.0 ± 3.2	67
Body mass (kg)	46.6 ± 18.0	116	45.2 ± 18.3	49	47.6 ± 17.9	67
Stature (cm)	147.5 ± 17.1	116	145.3 ± 16.0	49	149.0 ± 17.7	67
Body mass index Z-score	0.9 ± 1.1	116	0.8 ± 1.1	49	1.0 ± 1.2	67
Body mass index (categories, %)						
Underweight	3.5	4	6.1	3	1.5	1
Normoweight	61.2	71	65.4	32	58.2	39
Overweight	20.7	24	16.3	8	23.9	16
Obesity	14.6	17	12.2	6	16.4	11
Years from peak height velocity	−0.8 ± 2.7	116	0.0 ± 2.9	49	−1.3 ± 2.5	67
Cancer diagnoses (types, %)						
Acute lymphoblastic leukemia	38.8	45	36.7	18	40.3	27
Lymphoma	12.1	14	12.2	6	11.9	8
Central nervous system tumors	9.5	11	10.2	5	9.0	6
Renal tumors	7.8	9	4.1	2	10.5	7
Neuroblastoma	6.9	8	12.2	6	3.0	2
Malignant bone tumors	6.9	8	8.2	4	6.0	4
Histiocytosis	5.2	6	6.1	3	4.5	3
Soft tissue and other extraosseous sarcomas	4.3	5	0.0	0	7.5	5
Retinoblastoma	3.5	4	4.1	2	3.0	2
Hepatic tumors	2.6	3	4.1	2	1.5	1
Other malignant epithelial neoplasms	1.7	2	2.0	1	1.5	1
Unspecified malignant neoplasms	0.9	1	0.0	0	1.5	1
Cancer diagnoses (types, %)						
Blood	60.9	70	59.2	29	62.1	41
Solid	39.1	45	40.8	20	37.9	25
Time from treatment completion (years)	5.0 ± 3.8	113	5.2 ± 4.1	48	4.9 ± 3.6	65
Radiotherapy exposure (yes, %)	27.6	116	24.5	49	29.8	67
Fat mass Z-score	1.0 ± 0.9	116	0.8 ± 0.9	49	1.2 ± 0.9	67
Physical activity (min/day)	297.7 ± 84.0	110	298.1 ± 94.1	48	297.4 ± 76.0	62
Muscle strength deficits (%)	56.9	66	59.2	29	55.2	37
Low ALMI Z-score (%)	53.5	62	49.0	24	56.7	38
Sarcopenia Status (%)						
No Sarcopenia	43.1	50	40.8	20	44.8	30
Sarcopenia Probable	19.0	22	28.6	14	11.9	8
Sarcopenia Confirmed	37.9	44	30.6	15	43.3	29
HRQoL Z-score						
Total score	−0.9 ± 1.0	112	−1.0 ± 1.1	48	−0.9 ± 1.1	64
Physical health	−1.0 ± 1.0	112	−1.1 ± 1.1	48	−1.0 ± 1.1	64
Psychosocial health	−0.8 ± 1.0	112	−0.8 ± 1.0	48	−0.8 ± 1.0	64
Emotional functioning	−1.0 ± 1.0	112	−1.0 ± 1.1	48	−0.9 ± 1.0	64
Social functioning	−0.2 ± 0.9	112	−0.2 ± 0.9	48	−0.2 ± 1.0	64
School functioning	−0.8 ± 1.0	112	−0.7 ± 1.0	48	−0.8 ± 1.1	64

### Differences in HRQoL Z-score domains according to sarcopenia status

**Fig. 1:**
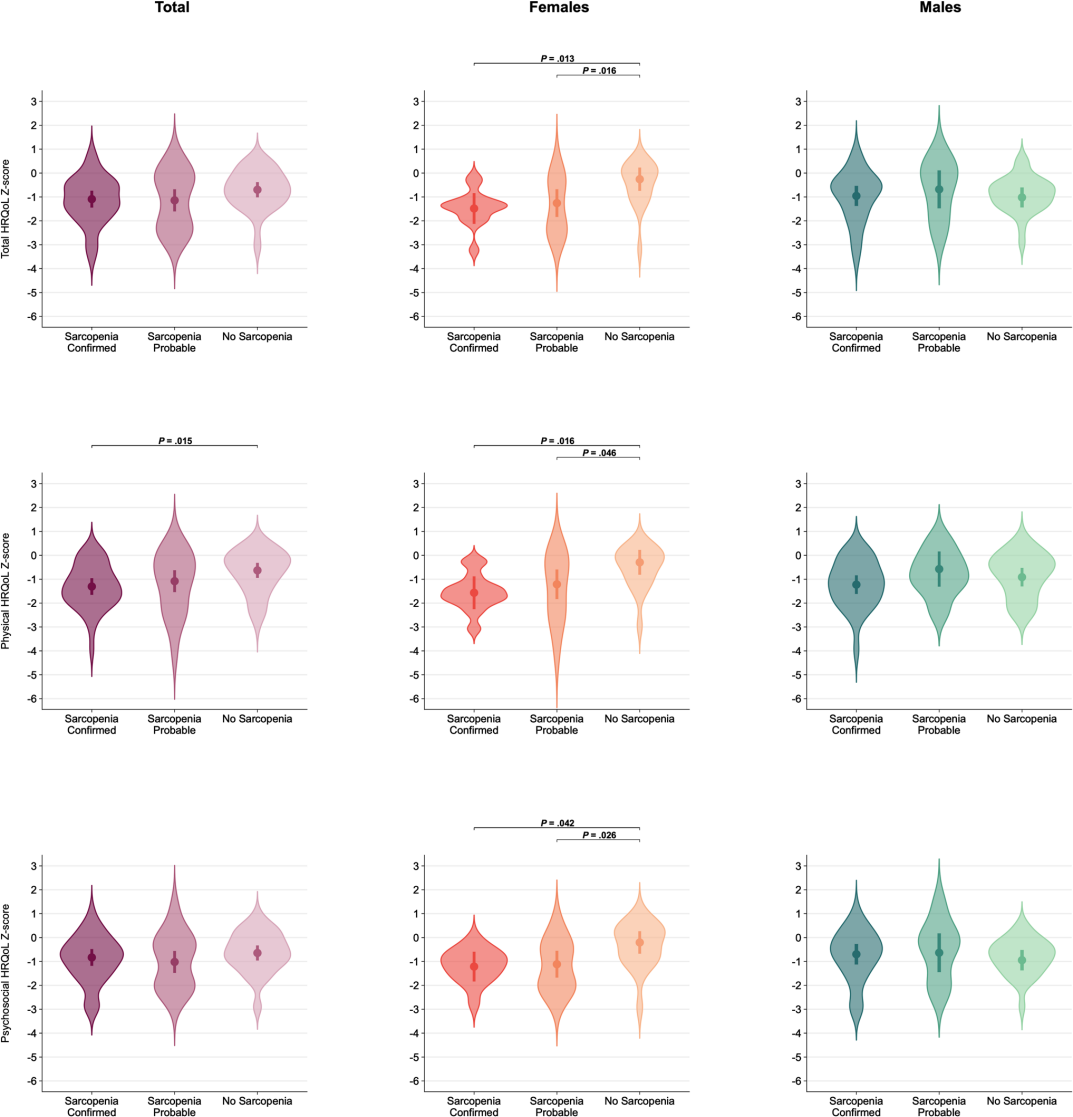
Differences in health-related quality of life (HRQoL) Z-score according to sarcopenia status in young pediatric cancer survivors. Data are presented as adjusted means and confidence intervals (95%). Violin plots show the distribution of the HRQoL domain within the sarcopenia status. Significant differences (adjusted P <.05) between sarcopenia status are shown in bold by analysis of covariance. Analyses were adjusted for time from treatment completion to baseline evaluation (years), radiotherapy exposure (yes/no), total body fat mass (Z-score) and total physical activity (min/day). Abbreviations: HRQoL = Health-related quality of life

Total HRQoL was similar across sarcopenia status for all survivors (Fig. 1). However, female survivors without sarcopenia had significantly better total HRQoL than female survivors with sarcopenia confirmed (−0.3 [95% CI: −0.8 to 0.2] vs. −1.5 [95% CI: −2.1 to −0.8]). In addition, female survivors without sarcopenia also had significantly better total HRQoL than female survivors with sarcopenia probable (−0.3 [95% CI: −0.8 to 0.2] vs. −1.3 [95% CI: −1.8 to −0.7]).

Physical HRQoL in survivors without sarcopenia was significantly better than in survivors with sarcopenia confirmed (−0.6 [95% CI: −1.0 to −0.3] vs. −1.3 [95% CI: −1.7 to −1.0]). Interestingly, female survivors without sarcopenia had significantly better physical HRQoL than female survivors with sarcopenia confirmed (−0.3 [95% CI: −0.8 to 0.2] vs. −1.6 [95% CI: −2.3 to −0.9]), being this difference lower in the total sample than in female survivors (0.7 vs. 1.3 [SD], respectively). Moreover, female survivors without sarcopenia also had significantly better physical HRQoL than female survivors with sarcopenia probable (−0.3 [95% CI: −0.8 to 0.2] vs. −1.2 [95% CI: −1.8 to −0.6]).

Psychosocial HRQoL was similar across sarcopenia status for all survivors. However, female survivors without sarcopenia had significantly better psychosocial HRQoL than female survivors with sarcopenia confirmed (−0.2 [95% CI: −0.7 to 0.3] vs. −1.2 [95% CI: −1.8 to −0.6]). In addition, female survivors without sarcopenia also had significantly better psychosocial HRQoL than female survivors with sarcopenia probable (−0.2 [95% CI: −0.7 to 0.3] vs. −1.1 [95% CI: −1.7 to −0.6]). There were no differences in HRQoL domains among males (Fig. 1).

**Fig. 2:**
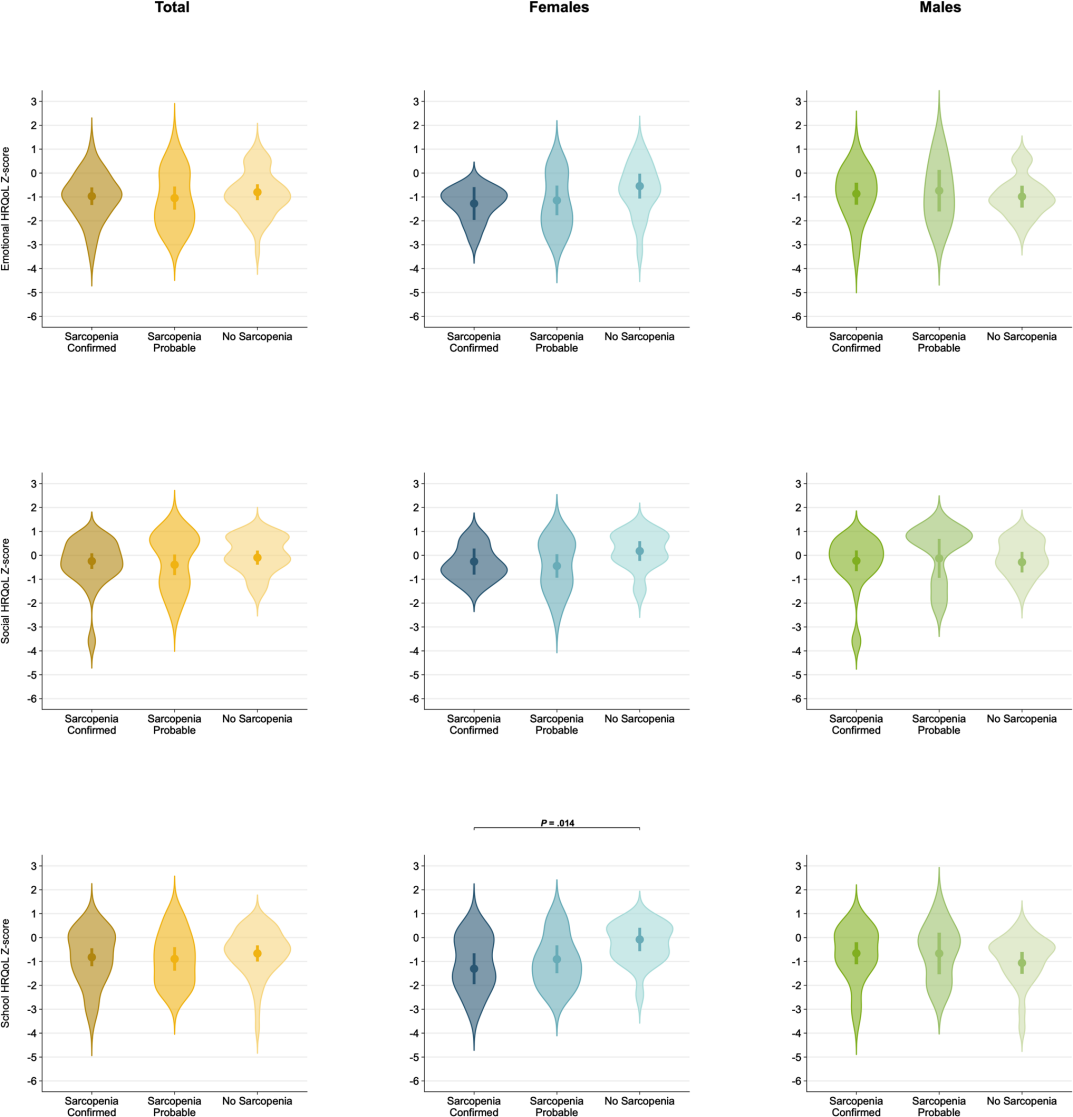
Differences in health-related quality of life (HRQoL) Z-score according to sarcopenia status in young pediatric cancer survivors. Data are presented as adjusted means and confidence intervals (95%). Violin plots show the distribution of the HRQoL domain within the sarcopenia status. Significant differences (adjusted P <.05) between sarcopenia status are shown in bold by analysis of covariance. Analyses were adjusted for time from treatment completion to baseline evaluation (years), radiotherapy exposure (yes/no), total body fat mass (Z-score) and total physical activity (min/day). Abbreviations: HRQoL = Health-related quality of life

Finally, emotional and social HRQoL were similar across sarcopenia status for all survivors, females and males (Fig. 2). School HRQoL was similar across sarcopenia status for all survivors, but females without sarcopenia had significantly better school HRQoL than females with sarcopenia confirmed (−0.1 [95% CI: −0.6 to 0.4] vs. −1.3 [95% CI: −2.0 to −0.7]). There were no differences in HRQoL domains among males (Fig. 2). Similar models adjusted for cancer diagnoses, instead of radiotherapy exposure, showed similar results across all HRQoL domains in both the total cohort and stratified by sex (Figures S3-4).

## Discussion

Most HRQoL domains are similar in young pediatric cancer survivors regardless of sarcopenia status. However, female survivors without sarcopenia have significantly better total, physical and school HRQoL Z-score than those with sarcopenia. There were no differences in HRQoL domains among male survivors by sarcopenia status. These results underline that females without sarcopenia report better overall HRQoL than females with sarcopenia after pediatric cancer.

The majority of previous studies [28, 34–37] investigating sarcopenia in young pediatric cancer survivors have followed different sarcopenia definitions excluding muscle strength, perhaps due to the young age of participants in some studies which makes it impossible to obtain reliable data at these ages [34, 37]. In our study, handgrip muscle strength and ALMI assessed by DXA were measured in accordance with the sarcopenia criteria established by the EWGSOP2, which has been previously applied in this population [28]. Our findings highlight the relevance of incorporating muscle strength (assessed by handgrip) into the definition of sarcopenia, as not all survivors with low ALMI exhibited deficits in muscle strength. The importance of including muscle strength in the definition of sarcopenia is supported by our findings as not all survivors in our study with low ALMI had muscle strength deficits. Furthermore, to the best of our knowledge, it remained unknown whether HRQoL could be better in young pediatric cancer survivors without sarcopenia than those with sarcopenia (including muscle strength deficits in addition to low ALMI).

Differences in HRQoL domains between female and male survivors were not detected. However, both reported lower HRQoL Z-scores than the normative reference values [31]. This is in line with a prior systematic review which evaluated the HRQoL of 14,342 pediatric cancer survivors in Europe who had survived at least five years after diagnosis [38]. Survivors reported worse HRQoL than not only normative data, but also comparison groups, yet differences were small to moderate. This offers clinicians valuable insight into potential complications that require attention as survival rates improve [1]. Investigating the factors that contribute to reduced HRQoL could support efforts to improve survivorship.

Contrary to prior reports in both adult cancer survivors [11] and adult pediatric cancer survivors [6], our findings showed no differences in most HRQoL domains across sarcopenia status in young pediatric cancer survivors, despite the presence of muscle strength deficits (56.9%), low lean mass (53.5%) and lower HRQoL within our cohort compared to normative data. Several factors may account for this discrepancy. First, the used HRQoL instrument (i.e., PedsQL 4.0 Generic Core Scales) may lack the sensitivity to detect subtle impairments in a clinical population such as our cohort of young pediatric cancer survivors, yet they reported lower HRQoL Z-scores than the normative reference values. Additionally, the relatively young age of our survivors may confer a greater degree of physiological resilience, potentially attenuating the negative relationship between sarcopenia and HRQoL that is observed later in life [39]. Moreover, the shorter time elapsed since treatment in our young population may limit the occurrence of long-term complications related to HRQoL. In this sense, psychosocial support systems commonly available to pediatric survivors, including family and school environments, may buffer the perceived impact of muscle strength and lean mass on overall HRQoL. Taken together, these findings suggest the need for longitudinal studies to better capture the evolving impact of sarcopenia on HRQoL over time. At this stage, sarcopenia may represent an early or subclinical marker of physical vulnerability that has not yet translated into perceivable limitations captured by generic HRQoL instruments.

The main finding of lower perceived burden of morbidity, as reflected in better total, physical and school HRQoL, for female pediatric cancer survivors without sarcopenia is of clinical interest. Prior investigations have shown that female pediatric cancer survivors are at higher risk of low HRQoL than male survivors [32]. Importantly, our study indicates that when female survivors do not have sarcopenia, their total, physical and school HRQoL is significantly better than when sarcopenia is present. There are very few reports describing the HRQoL of young pediatric cancer survivors with/without sarcopenia by sex. The observed sex differences may partly reflect reporting biases. For example, social norms around masculinity could influence how males perceive and report their physical and mental health, potentially leading to more favorable self-assessments of HRQoL. However, this interpretation should be considered speculative. Beyond psychosocial explanations, somatic maturity and hormonal factors may influence post-treatment muscle strength and lean mass trajectories, particularly given sex-specific differences during growth. Early exposure to aggressive cancer treatments (i.e., radiotherapy) may likely affect more younger survivors who have not reached somatic maturity peak than those older [6]. Differential fatigue profiles by sex may also contribute to recovery delays after treatment [40]. Additionally, body image disruption—especially in adolescent girls—may exacerbate the negative associations of sarcopenia with HRQoL. Future studies conducting separate analyses by sex are necessary to confirm our findings not only in female, but also in male survivors. In addition, physical activity interventions (i.e., resistance training) may help mitigate accelerated loss of muscle strength and lean mass (i.e., resistance training) and could have the potential of improving HRQoL, especially in female survivors with sarcopenia.

### Public health implications

The mounting body of evidence indicates that young pediatric cancer survivors are at higher risk of accelerated loss of lean mass and muscle strength [6, 41]. However, it remained unknown the associations of these early musculoskeletal limitations (sarcopenia) shortly after treatment completion on HRQoL of this population. Given that female pediatric cancer survivors specially suffer more from profound alterations in HRQoL [32], this study adds to the current literature that female survivors without sarcopenia have better total, physical and school HRQoL than female survivors with sarcopenia. These findings highlight the need for further investigation based on randomized controlled trials designed to improve lean muscle mass and strength, counteracting the premature arise of sarcopenia that could revert low HRQoL in young pediatric cancer survivors, especially in females.

### Limitations

The findings of this study need to be interpreted with some potential limitations. Firstly, yet sample size was calculated beforehand for the main aim of the iBoneFIT randomized controlled trial, some created categories in this cross-sectional study for comparisons across HRQoL domains by sarcopenia categories as well as sex-stratified analyses might be underpowered and can weaken the interpretability. Because we did not adjust for multiple comparisons in this exploratory analysis of secondary data, there is a possibility of type I error. Secondly, although we show results adjusted for major potential confounders identified through the DAG methodology (i.e., time from treatment completion, radiotherapy exposure, total body fat mass and total physical activity), residual confounding cannot be disregarded. Thirdly, survivors included in this study were those who opted to participate in an exercise intervention, and were not required to have neither sarcopenia nor low HRQoL since the main outcome of the iBoneFIT randomized controlled trial was bone health. In addition, they might have been more functional than the broader survivor population, likely resulting in underestimation of both sarcopenia prevalence and HRQoL impairments. Fourthly, the cross-sectional design does not allow to determine the causality of the findings and therefore, longitudinal studies are needed. Fifthly, sarcopenia conceptually includes muscle strength, and HRQoL also includes physical functioning domains and therefore, this conceptual overlap might inflate observed associations. Sixthly, there is currently no consensus definition of sarcopenia for pediatrics and hence, careful consideration should be applied when using the term sarcopenia in this study. Seventhly, differences in nutritional status, lean muscle mass and strength, and treatment protocols worldwide limit the generalizability of our findings. Eighthly, age- and sex-based reference values cannot fully substitute a prospectively recruited, well-matched control group assessed under identical conditions, particularly with respect to controlling for unmeasured confounders and methodological differences. Future studies would benefit from including matched healthy controls. Ninthly, survivors enrolled in this study (part of the iBoneFIT exercise intervention) might systematically be more functional than the broader survivor population, likely resulting in underestimation of both sarcopenia prevalence and HRQoL impairments. Finally, the heterogeneity of this study group of young pediatric cancer survivors should be considered when interpreting these findings. Future research should include larger and more homogeneous samples that enable a more detailed exploration of these differences.

## Conclusion

This study shows that most HRQoL domains are similar across sarcopenia status in young pediatric cancer survivors. However, female survivors without sarcopenia seem to have better total, physical and school HRQoL Z-score than those female survivors with sarcopenia. There were no differences in HRQoL domains among male survivors. These results underline that females without sarcopenia after pediatric cancer report better overall HRQoL than female survivors with sarcopenia. Additional research in larger cohort studies is necessary to confirm these findings and support their inclusion in future surveillance guidelines.

## Supplementary Information

Below is the link to the electronic supplementary material.

Supplementary file1 (DOCX 6822 KB)

## Data Availability

The datasets used and/or analysed in this study are available upon request.

## List of Abbreviations

ALM: Appendicular lean mass index.
CIS: Confidence intervals.
DAG: Directed acyclic graph.
EWGSOP2: European Working Group on Sarcopenia in Older People.
HRQoL: Health-related quality of life.
ICC: Intraclass correlation coefficients.
MSAS: Minimum sufficient adjustment set.
SD: Standard deviation.
STROBE: Strengthening the Reporting of Observational Studies in Epidemiology
