# Supplementary material for: Health-related quality of life in young pediatric cancer survivors: The role of sarcopenia
Source: Support Care Cancer. 2026 Jun 15;34(7):661. doi: 10.1007/s00520-026-10888-4 (PMC13269377; doi:10.1007/s00520-026-10888-4)
Supplement: Supplementary file 1 — Supplementary file1 (DOCX 6822 KB) [file 520_2026_10888_MOESM1_ESM.docx]

**Health-Related Quality of Life in Young Pediatric Cancer Survivors:**

**The Role of Sarcopenia**

***Supplementary material***

**
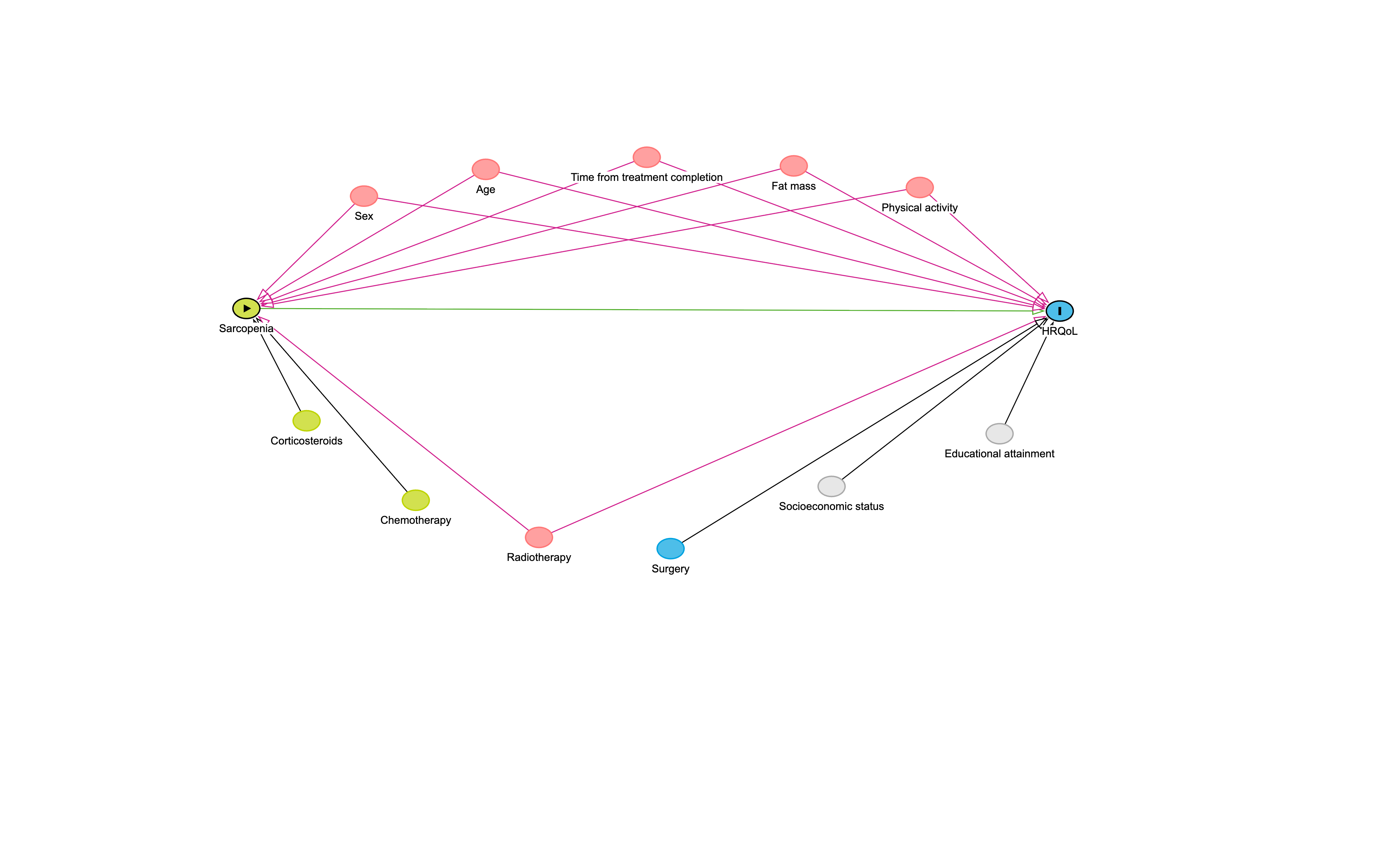
**

A

**
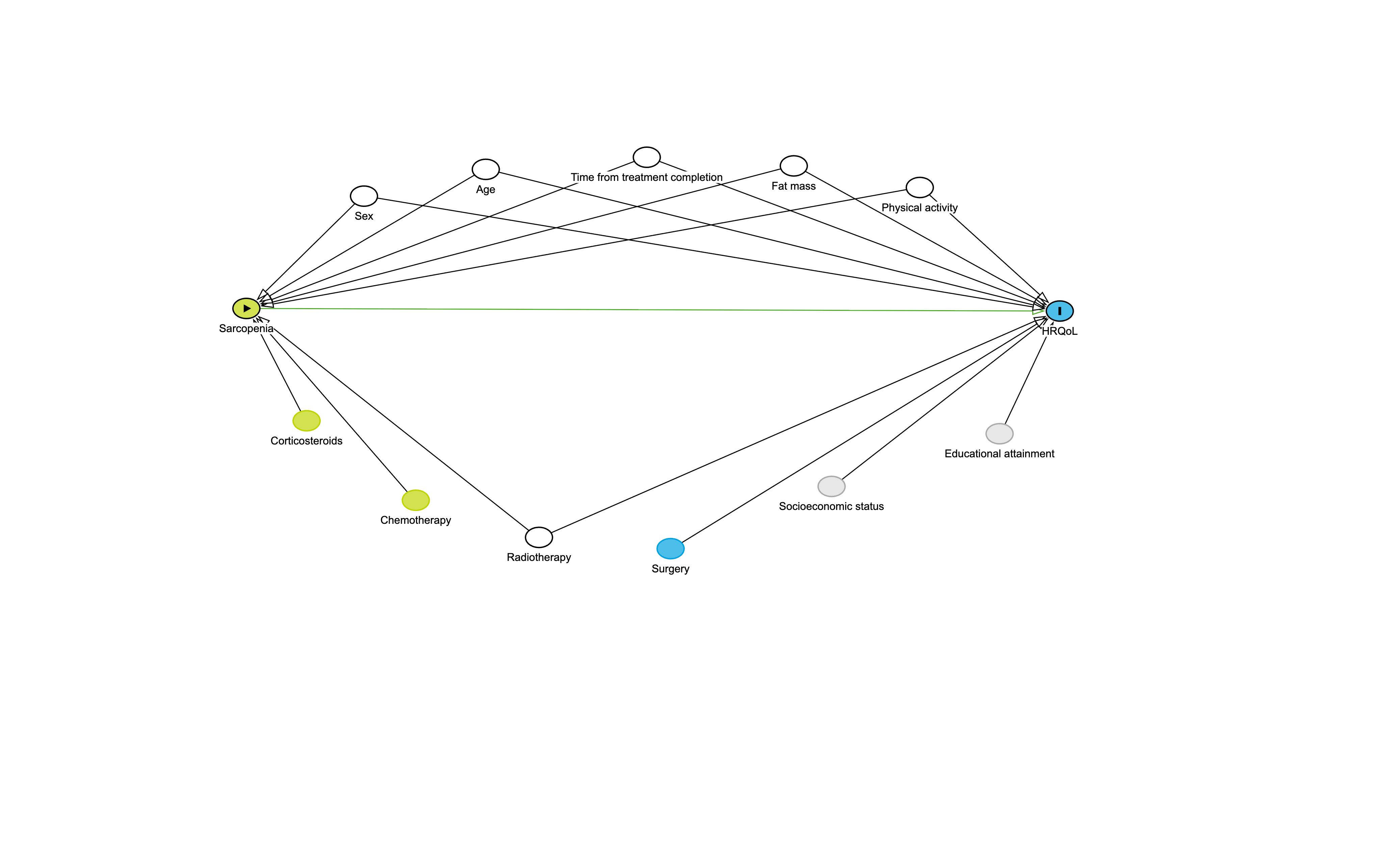
**

B

**Figure S1.** Directed acyclic graph (DAG).

**Panel A** represents the DAG for the causal structure of the relationship between sarcopenia (exposure, green circle) and HRQoL (outcome, blue circle). Pink circles indicate ancestor variables of both the exposure and the outcome (sex, age, time from treatment completion, total body fat mass, physical activity and radiotherapy exposure). Green circles indicate ancestor variables of the exposure (corticosteroids and chemotherapy). Blue circles indicate ancestor variables of the outcome (surgery, socioeconomic status and educational attainment). Green arrows indicate "causal" paths, and pink arrows indicate biasing paths.

**Panel B** represent the DAG after adjusting for the minimum sufficient adjustment set for the total effect (i.e., sex, age, time from treatment completion, total body fat mass, physical activity and radiotherapy exposure, now represented with white circles). Note that the biasing paths were completely closed (pink arrows became black arrows, suggesting the correct control for the relevant confounders), and that only the "causal" paths remained opened (both the direct path and the indirect paths, i.e., through mediators).

Abbreviations: HRQoL = Health-related quality of life.





**Figure S2.** Flow chart.

**
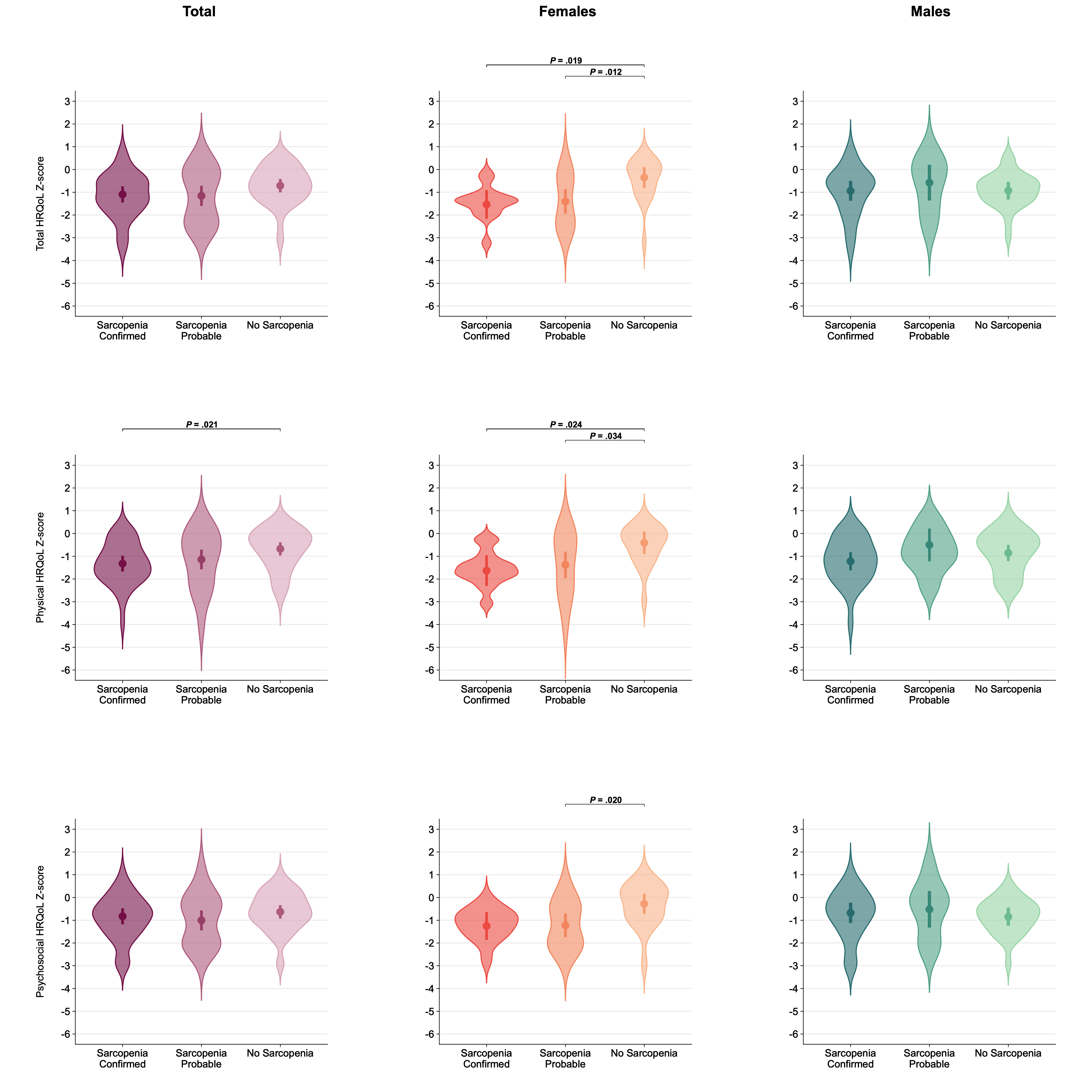
**

**Figure S3.** Differences in health-related quality of life (HRQoL) Z-score according to sarcopenia status in young pediatric cancer survivors. Data are presented as adjusted means and confidence intervals (95%). Violin plots show the distribution of the HRQoL domain within the sarcopenia status. Significant differences (adjusted P < .05) between sarcopenia status are shown in bold by analysis of covariance. Analyses were adjusted for time from treatment completion to baseline evaluation (years), cancer diagnosis (blood/solid), total body fat mass (Z-score) and total physical activity (min/day). Abbreviations: HRQoL = Health-related quality of life.


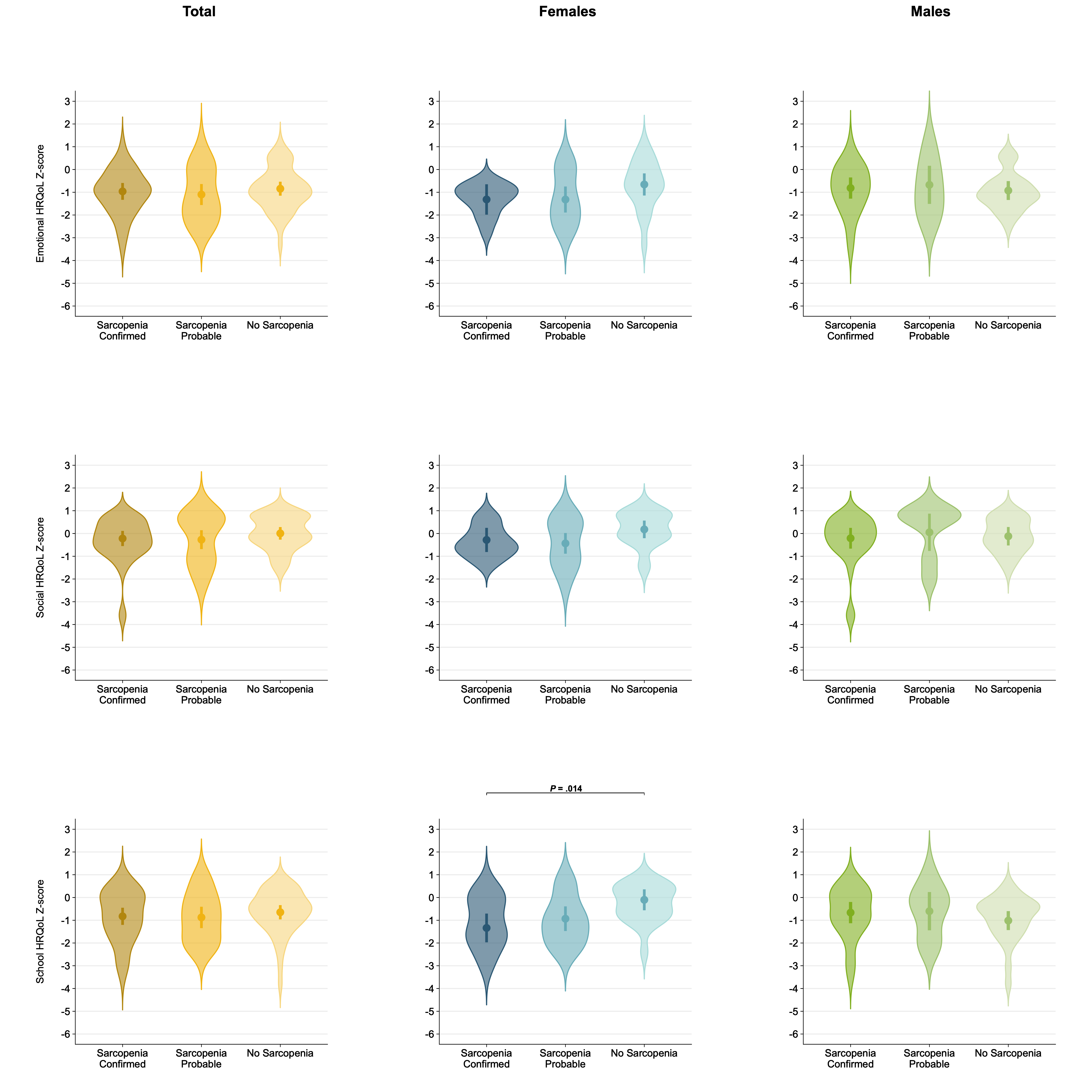


**Figure S4.** Differences in health-related quality of life (HRQoL) Z-score according to sarcopenia status in young pediatric cancer survivors. Data are presented as adjusted means and confidence intervals (95%). Violin plots show the distribution of the HRQoL domain within the sarcopenia status. Significant differences (adjusted P < .05) between sarcopenia status are shown in bold by analysis of covariance. Analyses were adjusted for time from treatment completion to baseline evaluation (years), cancer diagnosis (blood/solid), total body fat mass (Z-score) and total physical activity (min/day). Abbreviations: HRQoL = Health-related quality of life.

**Table S1.** STROBE Statement - Checklist of items that should be included in reports of cross-sectional studies.

|  | Item No | Recommendation | Page No |
| --- | --- | --- | --- |
| Title and abstract | 1 | (*a*) Indicate the study’s design with a commonly used term in the title or the abstract | 1 |
|  |  | (*b*) Provide in the abstract an informative and balanced summary of what was done and what was found | 3 |
| Introduction | | | |
| Background/rationale | 2 | Explain the scientific background and rationale for the investigation being reported | 5 |
| Objectives | 3 | State specific objectives, including any prespecified hypotheses | 5 |
| Methods | | | |
| Study design | 4 | Present key elements of study design early in the paper | 5-6 |
| Setting | 5 | Describe the setting, locations, and relevant dates, including periods of recruitment, exposure, follow-up, and data collection | 5-6 |
| Participants | 6 | (*a*) Give the eligibility criteria, and the sources and methods of selection of participants | 5-6 |
| Variables | 7 | Clearly define all outcomes, exposures, predictors, potential confounders, and effect modifiers. Give diagnostic criteria, if applicable | 6-9 |
| Data sources/ measurement | 8 | For each variable of interest, give sources of data and details of methods of assessment (measurement). Describe comparability of assessment methods if there is more than one group | 6-9 |
| Bias | 9 | Describe any efforts to address potential sources of bias | 9 |
| Study size | 10 | Explain how the study size was arrived at | 5-6 |
| Quantitative variables | 11 | Explain how quantitative variables were handled in the analyses. If applicable, describe which groupings were chosen and why | 9 |
| Statistical methods | 12 | (*a*) Describe all statistical methods, including those used to control for confounding | 9 |
|  |  | (*b*) Describe any methods used to examine subgroups and interactions | 9 |
|  |  | (*c*) Explain how missing data were addressed | 9 |
|  |  | (*d*) If applicable, describe analytical methods taking account of sampling strategy | 9 |
|  |  | (*e*) Describe any sensitivity analyses | 9 |
| Results | | | |
| Participants | 13 | (a) Report numbers of individuals at each stage of study—eg numbers potentially eligible, examined for eligibility, confirmed eligible, included in the study, completing follow-up, and analysed | 9-10 |
|  |  | (b) Give reasons for non-participation at each stage | 9-10 |
|  |  | (c) Consider use of a flow diagram | 9-10 |
| Descriptive data | 14 | (a) Give characteristics of study participants (eg demographic, clinical, social) and information on exposures and potential confounders | Table 1 and Table S2 |
|  |  | (b) Indicate number of participants with missing data for each variable of interest | Table 1 |
| Outcome data | 15 | Report numbers of outcome events or summary measures | Table 1 |
| Main results | 16 | (*a*) Give unadjusted estimates and, if applicable, confounder-adjusted estimates and their precision (eg, 95% confidence interval). Make clear which confounders were adjusted for and why they were included | Figure 1-2 |
|  |  | (*b*) Report category boundaries when continuous variables were categorized | Not applicable |
|  |  | (*c*) If relevant, consider translating estimates of relative risk into absolute risk for a meaningful time period | Not applicable |
| Other analyses | 17 | Report other analyses done—eg analyses of subgroups and interactions, and sensitivity analyses | 9-10 |
| Discussion | | | |
| Key results | 18 | Summarise key results with reference to study objectives | 10-11 |
| Limitations | 19 | Discuss limitations of the study, taking into account sources of potential bias or imprecision. Discuss both direction and magnitude of any potential bias | 13 |
| Interpretation | 20 | Give a cautious overall interpretation of results considering objectives, limitations, multiplicity of analyses, results from similar studies, and other relevant evidence | 10-14 |
| Generalisability | 21 | Discuss the generalisability (external validity) of the study results | 14 |
| Other information | | | |
| Funding | 22 | Give the source of funding and the role of the funders for the present study and, if applicable, for the original study on which the present article is based | 15 |

Note: An Explanation and Elaboration article discusses each checklist item and gives methodological background and published examples of transparent reporting. The STROBE checklist is best used in conjunction with this article (freely available on the Web sites of PLoS Medicine at http://www.plosmedicine.org/, Annals of Internal Medicine at http://www.annals.org/, and Epidemiology at http://www.epidem.com/). Information on the STROBE Initiative is available at [www.strobe-statement.org](http://www.strobe-statement.org).

**Table S2.** Bivariate correlation coefficients between cancer treatment, sarcopenia and HRQoL Z-score domains in young paediatric cancer survivors.

|  | Radiotherapy  exposure | Chemotherapy  exposure | Corticosteroids exposure | Surgery |
| --- | --- | --- | --- | --- |
| Sarcopenia status |  |  |  |  |
| Muscle strength | 0.146 | 0.132 | -0.141 | -0.116 |
| ALMI Z-score | -0.240** | 0.086 | 0.271** | -0.165 |
| HRQoL Z-score |  |  |  |  |
| Total score | -0.110 | 0.170 | 0.165 | -0.104 |
| Physical health | -0.085 | 0.067 | 0.149 | -0.093 |
| Psychosocial health | -0.117 | 0.201* | 0.147 | -0.094 |
| Emotional functioning | 0.007 | 0.035 | 0.033 | -0.016 |
| Social functioning | -0.225* | 0.284** | 0.148 | -0.059 |
| School functioning | -0.122 | 0.197* | 0.134 | -0.102 |

Abbreviations: MD, mean difference. ** denotes significant at the 0.01 level (2-tailed) and * denotes significant at the 0.05 level (2-tailed).
